# Reality Monitoring and Metamemory in Adults with Autism Spectrum Conditions

**DOI:** 10.1007/s10803-016-2749-x

**Published:** 2016-02-22

**Authors:** Rose A. Cooper, Kate C. Plaisted-Grant, Simon Baron-Cohen, Jon S. Simons

**Affiliations:** Department of Psychology, University of Cambridge, Downing Street, Cambridge, CB2 3EB UK; Autism Research Centre, Department of Psychiatry, University of Cambridge, Cambridge, UK

**Keywords:** Autism, Episodic memory, Reality monitoring, Metacognition, Metamemory

## Abstract

Studies of reality monitoring (RM) often implicate medial prefrontal cortex (mPFC) in distinguishing internal and external information, a region linked to autism-related deficits in social and self-referential information processing, executive function, and memory. This study used two RM conditions (self-other; perceived-imagined) to investigate RM and metamemory in adults with autism. The autism group showed a deficit in RM, which did not differ across source conditions, and both groups exhibited a self-encoding benefit on recognition and source memory. Metamemory for perceived-imagined information, but not for self-other information, was significantly lower in the autism group. Therefore, reality monitoring and metamemory, sensitive to mPFC function, appear impaired in autism, highlighting a difficulty in remembering and monitoring internal and external details of past events.

## Introduction

### Reality Monitoring and the mPFC

Episodic memory is the ability to recall details of a specific event, such as temporal, visuo-spatial, and cognitive information (Tulving 1985), while source memory specifically refers to memory for the specific context in which an event was experienced, facilitated by source monitoring processes that evaluate memory characteristics and facilitate the source memory decision (Johnson et al. 1993). For example, discriminating between internal and external sources of information is referred to as ‘reality monitoring’ (Johnson et al. 1993), where internally-generated memories are likely to contain more cognitive operations (e.g. thoughts) than externally-generated memories, which in turn are more likely to contain a greater number of perceptual details (Johnson and Raye 1981). Source monitoring processes can either be relatively automatic or more strategic if, for example, two sources are quite similar in nature.

The prefrontal cortex (PFC) has been widely implicated in source memory, with evidence from lesion patients and functional neuroimaging converging to support a role of the PFC in the retrieval of a range of source contexts (Dobbins et al. 2002; Duarte et al. 2005; Simons et al. 2002, 2005; Turner et al. 2008). However, the medial PFC (mPFC) appears to be particularly sensitive to the dissociation between internal and external sources, such as perceived and imagined contexts, compared to other types of source judgements (Brandt et al. 2014; Simons et al. 2006; Turner et al. 2008). Simons et al. (2008) investigated the neural basis of memory for two different forms of internal–external information: ‘self’- or ‘other’-generated information and ‘perceived’ or ‘imagined’ information. Interestingly, a relatively caudal mPFC region showed significantly greater activity during discrimination of self-other relative to perceived-imagined sources, with the latter being associated with more rostral mPFC activity, highlighting functional specialization despite highly overlapping activity associated with source retrieval.

The region of the mPFC identified by Simons et al. (2008) during the self-other discrimination consistently exhibits activity during mentalizing (Gilbert et al. 2006), an ability to consider different perspectives of ourselves and others. The mPFC is thought to play a central role in reasoning about the self and others (Amodio and Frith 2006; Buckner and Carroll 2007; Saxe et al. 2006), with evidence supporting the relationship between mPFC activity and distinguishing between objects processed in relation to oneself or someone else (Kim and Johnson 2012). This region has also been associated with memory for self-related information (Bergstrom et al. 2015; Zhu et al. 2012) and with the benefit of self-referential encoding on source memory (Leshikar and Duarte 2013). Conversely, the more rostral region of the mPFC identified by Simons et al. (2008), active during perceived-imagined reality monitoring, is involved in multi-task coordination (Gilbert et al. 2006), in line with evidence that the rostral mPFC is sensitive to switching between perceptual and cognitive decisions (Gilbert et al. 2005). The functional distinction within mPFC has been supported by Gilbert et al. (2007), who observed caudal mPFC activity for mentalizing versus non-mentalizing tasks and rostral mPFC activity during perception versus imagining, leading to the suggestion that the rostral mPFC is involved in monitoring internal and external processes and attention switching. Such processes also contribute to metamemory, monitoring the accuracy of one’s memory, which has been linked with mPFC function (Baird et al. 2013; Do Lam et al. 2012; see Fleming and Dolan 2012 for a review), further supporting the importance of the mPFC for monitoring the internal and external details of our memories.

### Memory and Reality Monitoring in Autism Spectrum Conditions

Autism spectrum conditions (henceforth, autism) are associated with deficits in social and self-referential information processing (Lombardo and Baron-Cohen 2011; Frith 2001; Williams 2010) and much neurological evidence points to the mPFC as an important site of dysfunction underpinning these characteristics (Ben Shalom 2009; Uddin 2011). Reduced mPFC activity has been reported in individuals with autism during tasks requiring mentalizing (Frith 2001; Murdaugh et al. 2012; White et al. 2014), with mPFC activity levels distinguishing less between ‘self’ and ‘other’ during self-reference and self-other judgement tasks than in typical individuals (Kennedy and Courchesne 2008; Lombardo et al. 2009), suggesting that representations of self- and other-related information may not be as distinct.

Subtle memory deficits also exist in autism, largely consisting of impaired episodic memory but intact semantic memory (Boucher et al. 2012; Bowler et al. 2011). Impaired episodic memory in autism has been suggested to result from mPFC dysfunction (Brezis 2015), influenced by deficits in mentalizing (Baron-Cohen 1995) and self-projection (Lind 2010; Lind et al. 2014), and some evidence has suggested disproportionate deficits in monitoring and retrieving information regarding the self and others. For instance, individuals with autism have impaired recollection of social details relative to other perceptual details (O’Shea et al. 2005) and reduced memory for socially-encoded words (Brezis et al. 2013), as well as a specific reduction in recollection of socially salient aspects of scenes (Bruck et al. 2007). With regard to the ‘self’, individuals with autism exhibit a reduced self-reference effect in memory (Grisdale et al. 2014; Henderson et al. 2009; Lombardo et al. 2007), and episodic memory in autism is less organised around self goals (Crane et al. 2009) and is less likely to be retrieved from a first-person perspective (Lind and Bowler 2010; Lind et al. 2014). However, episodic memory deficits involving non-social or non-self oriented stimuli (e.g. Bowler et al. 2007, 2014; Cooper et al. 2015) call into question whether memory deficits in autism are solely characterised by mentalizing and self-reference deficits. Investigating reality monitoring in autism may thus provide valuable insights to resolve this question.

However, findings from reality monitoring studies in autism have been inconsistent and have largely focused on self-other source memory alone. Some studies have observed an impairment in the ability of individuals with autism to recollect whether they or someone else performed an action (Lind and Bowler 2009; Maras et al. 2013; Russell and Jarrold 1999), whereas other studies have reported no difference in self-other reality monitoring ability (Farrant et al. 1998; Grainger et al. 2014a; Hill and Russell 2002; Zalla et al. 2010). Across most of these studies, the number of participants and trials has been small, limiting the power to uncover subtle differences. It is interesting to note that the studies with the most trials (Maras et al. 2013) and most participants (Lind and Bowler 2009) both observed deficits in self-other source memory. However, these studies only examined one type of reality monitoring and cannot determine whether a deficit in processing information in relation to the self and others is specifically responsible for the reality monitoring impairment. Only one study has compared reality monitoring of self-other and perceived-imagined sources in children with autism, demonstrating a deficit across both conditions (Hala et al. 2005). Hala et al. interpreted their results as supporting an executive function framework, as a primary mentalizing deficit would have predicted a disproportionate reduction in self-other reality monitoring. Therefore, reality monitoring differences in autism may not be solely driven by difficulties processing information about the self and others, but may be also influenced by the monitoring demands of the task.

The findings of Hala et al. could possibly be explained by evidence of atypical mPFC activity during switching between internal and external information in individuals with autism (Gilbert et al. 2008). Furthermore, Gilbert et al. (2009) compared an internal-external attention orienting task and a mentalizing task, finding that individuals with autism showed a distinct lack of neural functional specialization between the tasks, which could lead to generalised rather than specific reality monitoring impairments. Consistent with Hala et al.’s suggestion of monitoring and attention switching influences on memory deficits in autism, recent evidence has demonstrated a strong relationship between executive function and episodic memory in these individuals (Goddard et al. 2014; Maister et al. 2013). Additionally, the benefit of task support, such as providing retrieval cues to support memory retrieval, on recall and source memory in autism (Bowler et al. 2004; Maras et al. 2013) further highlights the influence of retrieval monitoring demands on memory deficits. Specifically, it is believed that memory impairments in autism increase as the complexity of the task demands increase (Minshew and Goldstein 2001); suggesting that monitoring and attention switching requirements during retrieval may influence deficits seen in source recall and episodic memory. Direct evidence for monitoring impairments during memory tasks in autism comes from studies showing a reduction in metacognition, specifically, impaired metamemory as shown by less accurate ‘feeling of knowing’ judgements (Grainger et al. 2014b; Wojcik et al. 2013) and a reduced relationship between confidence and recognition memory (Wilkinson et al. 2010). These findings suggest that a deficit in autism in distinguishing between internal and external sources of information in memory might also extend to an impairment in monitoring the accuracy of these source memory decisions, which has yet to be investigated.

The aim of the current study is to investigate the pattern of reality monitoring and metamemory impairments in adults with autism, due to known mPFC dysfunction in this population and the role of this region in reality monitoring and metamemory, to compare the influence of self/social information processing with monitoring and switching between internal and external processes on memory in autism. We adapted the task used by Simons et al. (2008) to allow us to assess recognition memory as well as source memory and memory confidence. The task tests participants’ ability to discriminate between self-other and perceived-imagined sources in memory and to monitor the accuracy of these source memory decisions. This task has increased sensitivity relative to previous studies and allowed us to assess each kind of source memory within the same task, thereby controlling for any extraneous processes that may have influenced the findings of previous studies examining one type of source alone. The reality monitoring task has been used in a number of previous studies, exhibiting sensitivity to individual differences in typical adults (Simons et al. 2006, 2008; Gilbert et al. 2010; Buda et al. 2011), and in individuals with proneness to or risk of developing psychosis (Lagioia et al. 2011; Simons et al. 2008). We aimed to test whether the ‘self’ has a reduced benefit on recognition memory and source memory in autism, and whether memory for self-other sources might be disproportionately impaired in adults with autism due to a reduction in mentalizing, or whether discriminating between perceived and imagined sources might also be impaired, reflecting a more general deficit in monitoring information in memory. To this end, we also assessed metacognitive sensitivity to test whether metamemory deficits extend to source memory in autism.

## Method

### Participants

Twenty-four participants with a diagnosis of autism (13 females, 11 males) and twenty-four control participants (13 females, 11 males) took part. All participants were aged between 18 and 45, and had normal or corrected-to-normal vision and hearing. No participant in the control group had a known current or historical diagnosis of any psychiatric, neurological or developmental condition. Participants in the autism group had a formal diagnosis of high-functioning autism (N = 2) or Asperger Syndrome (N = 22) according to DSM-5 (American Psychiatric Association 2013) or ICD-10 criteria, and received their diagnosis following specialist assessment by a qualified clinician. All participants were administered the Autism Spectrum Quotient (AQ; Baron-Cohen et al. 2001), the short-form Raven’s advanced progressive matrices (Arthur and Day 1994), the WAIS-III vocabulary test (Wechsler 1997), and semantic and phonological fluency tests. The AQ is a 50 item questionnaire measuring self-reported autistic traits, the short-form Raven’s Matrices assesses non-verbal abstract reasoning to complete 12 items, yielding a maximum score of 12. The WAIS vocabulary test requires participants to define a series of 33 words, with a maximum score of 66, and the semantic and phonological verbal fluency tests requires participants to generate as many words as possible beginning with the letter ‘b’ or associated with the category ‘animals’, respectively, in 90 s. The WAIS vocabulary test and Raven’s matrices were chosen as short but reliable measures of verbal and non-verbal ability, and the verbal fluency test was administered as a control because the memory task used involved generating words. The groups were matched on age, years of education, verbal and non-verbal ability, and phonological and semantic fluency (all *p* > .33; see Table 1), and the autism group scored significantly higher on the AQ than the control group (t(46) = 12.91, *p* < .001).

**Table 1:** Demographic information and psychometric test scores within each group: mean (std)

	Autism (N = 24)	Control (N = 24)
Age	31.38 (7.28)	30.46 (6.95)
Years of education	15.50 (2.13)	15.96 (2.14)
AQ	37.38 (7.03)	14.54 (5.07)
Raven’s percentile^a^	75.42 (27.66)	70.83 (29.25)
WAIS vocabulary^b^	13.29 (2.37)	13.13 (2.72)
Phonological fluency	26.58 (7.79)	27.67 (5.05)
Semantic fluency	35.83 (9.61)	38.33 (8.17)

^a^Raven’s score are standardised

^b^WAIS scores are standardised

Participants with autism were recruited from a participant database held by the Cambridge Laboratory for Research into Autism, and the Cambridge Autism Research Centre’s participant database. Control participants were recruited via an existing participant database maintained by the Behavioural and Clinical Neuroscience Institute (BCNI), Cambridge University, as well as via social media adverts. Ethical approval for this study was obtained from the Cambridge Psychology Research Ethics Committee. Participants gave written informed consent prior to taking part and were paid a standard honorarium for their time.

### Design and Procedure

**Fig. 1:**
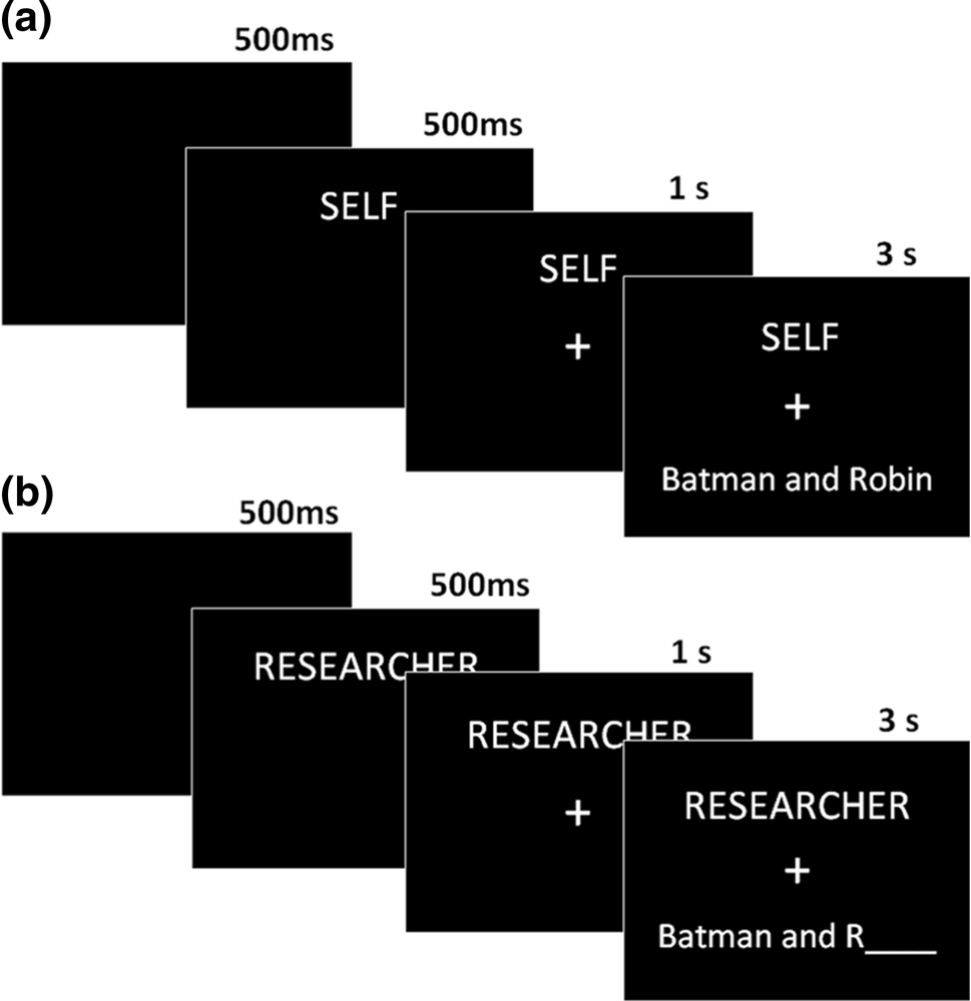
An example of trials in the SELF-PERCEIVED study condition (**a**) and the RESEARCHER-IMAGINED study condition (**b**)

The computer-based reality monitoring task included 144 study phase trials and 216 test phase trials (including the studied stimuli and 72 new stimuli) divided into 6 study-test blocks. The stimuli consisted of common word pairs (e.g. “Batman and Robin”), collated from previous studies (Simons et al. 2006, 2008; Buda et al. 2011) that extensively piloted the word-pairs to ensure their familiarity (see “Appendix”). The word-pairs were studied in one of four encoding conditions: ‘self-perceived (SP)’, ‘self-imagined’ (SI), ‘experimenter-perceived’ (EP), and ‘experimenter-imagined’ (EI), with 36 word-pairs per condition. For ‘self’ word-pairs, participants were instructed to read the word-pair out loud and, for ‘experimenter’ word-pairs, they were informed that the experimenter would read the word-pair out loud. ‘Perceived’ trials were those in which both words in the word-pair were shown on the screen and ‘imagined’ word-pairs were trials in which just the first word and the first letter of the second word were displayed (e.g. “Batman and R____”) and the participant or experimenter had to imagine the second word in the pair before saying the word-pair aloud (see Fig. 1). If the participant struggled to complete the word-pair in an ‘imagine’ trial then they were encouraged to generate and speak aloud a suitable guess.

**Fig. 2:**
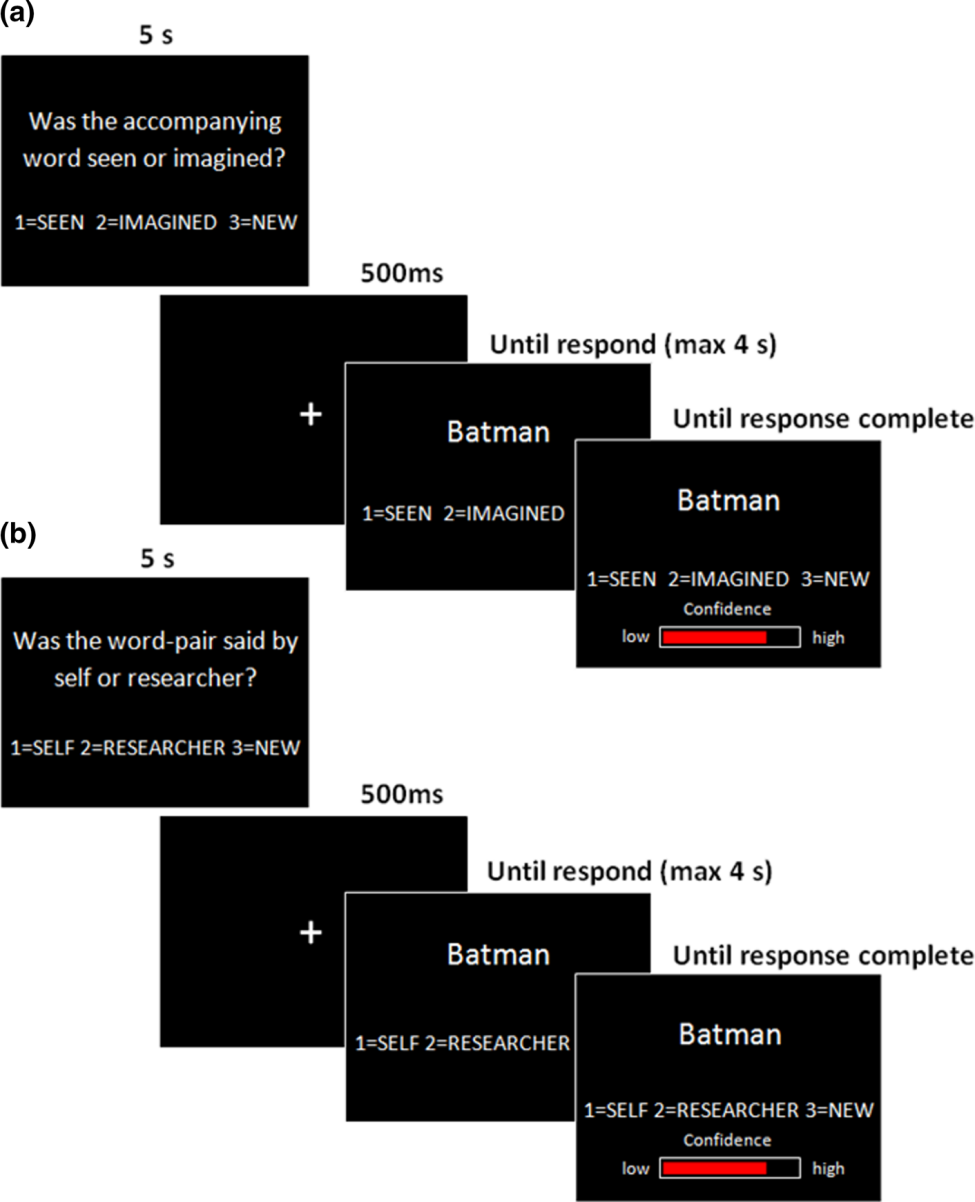
An example of a trial in the PERCEIVED/IMAGINED test condition (**a**) and an example of a trial in the SELF/EXPERIMENTER test condition (**b**)

Each test phase was completed immediately after studying all 24 word-pairs in the block. Participants were tested on the first word from each of the studied word-pairs or the first word from a new, unstudied, word-pair. For half of all 36 words tested per block, they were tested on their memory for whether the corresponding word-pair had been said by ‘self’ or the ‘researcher’ during the study phase or if the word was new (‘Self/Experimenter’ or ‘SE’ condition), and, for the other half, if the second word of the word-pair had been ‘seen’ or ‘imagined’ during the study phase or if the word was new (‘Perceived/Imagined’ or ‘PI’ condition) (see Fig. 2). Therefore, of all 144 studied words, 72 were tested in the SE condition and 72 were tested in the PI condition, with each test condition including 18 word-pairs from each of the four encoding conditions. Both accuracy and time taken to respond were measured. For each word, participants indicated their confidence on a continuous scale of ‘low’ to ‘high’. Confidence was determined by the duration the participant held down their response key to move a bar on the screen from low to high (range 0–1000 ms). Participants were instructed to think about how confident they were in each of their responses and to use the whole confidence range accordingly throughout the task. The order of the SE and PI test conditions was counterbalanced across the 6 blocks. Presentation of the word pairs as old or new was counterbalanced, as was studying the word-pairs in each of the four study conditions and testing the word-pairs in either the SE or PI condition. Trials were pseudorandomised so that no more than three trials in a row were from the same condition for both study and test phases. Participants were given an instruction sheet and completed a practice task before starting the experiment.

After the reality monitoring task, participants completed a debriefing questionnaire and the AQ. Participants then completed the Raven’s matrices followed by the verbal fluency tasks and the WAIS vocabulary sub-test. The tasks were completed in this order for every participant and the total testing session lasted up to 1 ½ hours.

### Data Analysis

All analyses were conducted using two-tailed tests at a standard alpha level of .05. Effect sizes are reported using eta-squared (η^2^) values for analyses of variance (ANOVAs) and Cohen’s *d* for *t* tests. First, overall recognition memory during both the SE and PI test conditions was assessed, and it was then tested whether recognition memory was affected by encoding condition and if this differed between groups. Analyses of source memory used a conditional measure of source accuracy, defined as the proportion of correct source responses for word-pairs correctly recognised. Analyses assessed overall source memory in the SE and PI test conditions, accuracy for each source within the SE and PI test conditions (S vs E; P vs I, respectively), and then to see how source memory accuracy in the SE and PI test conditions is affected by encoding condition (P vs I; S vs E, respectively). Analyses then focused on source metamemory, defined as the trial-by-trial correlation between source memory accuracy (0, 1) and confidence (0–1000). This measure of metamemory was chosen due to the continuous nature of the confidence response and to maximise sensitivity to detect subtle differences in metacognition over and above discrete ratings.

## Results

### Recognition

To assess recognition memory accuracy, *d*’ was calculated for both the SE and PI test conditions. Recognition ‘hits’ were defined as the percentage of studied words correctly identified as old regardless of the source the participant chose, and false alarms (FAs) were defined as the proportion of new items misattributed to one of the two sources. A 2 group (autism, control) × 2 test condition (SE, PI) ANOVA on recognition *d*’ revealed no main effects or interaction between factors (Fs < .2, *p*s > .7, *η*^2^ < .01), demonstrating that recognition *d*’ did not differ between the autism (mean = 2.89, std = 0.53) and control (mean = 2.85, std = 0.62) groups. The proportion of studied words correctly recognised was high in both the SE (autism: mean = 0.87, std = 0.08; control: mean = 0.88, std = 0.06) and PI (autism: mean = 0.87, std = 0.09; control: mean = 0.89, std = 0.06) test conditions. *T* tests performed on confidence ratings and time taken to correctly reject new words also showed no difference between the groups (ts < 1, *p*s > .5, *d*s < 0.17). Therefore, recognition memory of the autism and control groups was very similar overall.

#### Effect of Encoding Condition on Recognition

To investigate the effect of encoding condition on subsequent recognition memory, an ANOVA was conducted on recognition of words studied in each of the four encoding conditions using a 2 (S, E) × 2 (P, I) × 2 (autism group, control group) analysis (see Table 2 for mean recognition accuracy by encoding condition). A self-reference effect was observed as the proportion of recognised words was significantly higher for previously self-spoken items than for experimenter items, F(1,46) = 53.65, *p* < .001, *η*^2^ = .28, and this effect did not differ between the groups (F < .2, *p* > .7, *η*^2^ < .01). A significant generation effect, a benefit of imagining items on later recognition, was also observed, F(1,46) = 97.29, *p* < .001, *η*^2^ = .30, which did not differ between the groups (F < .1, *p* > .8, *η*^2^ < .01). There was no significant interaction between SE and PI (F = 2.3, *p* = .13, *η*^2^ = .01), which did not vary by group (F = 0.0, *p* > .9, *η*^2^ < .01). Therefore, recognition memory of the autism and control groups was similarly affected by encoding condition.

**Table 2:** d’ the proportion of correctly recognised words that were studied in each of the four encoding conditions (self-perceived, self-imagined, experimenter-perceived, experimenter-imagined): mean (std)

	SP	SI	EP	EI
Control	0.90 (0.09)	0.97 (0.03)	0.78 (0.09)	0.89 (0.09)
Autism	0.88 (0.10)	0.95 (0.06)	0.77 (0.13)	0.88 (0.13)

### Source Memory

**Fig. 3:**
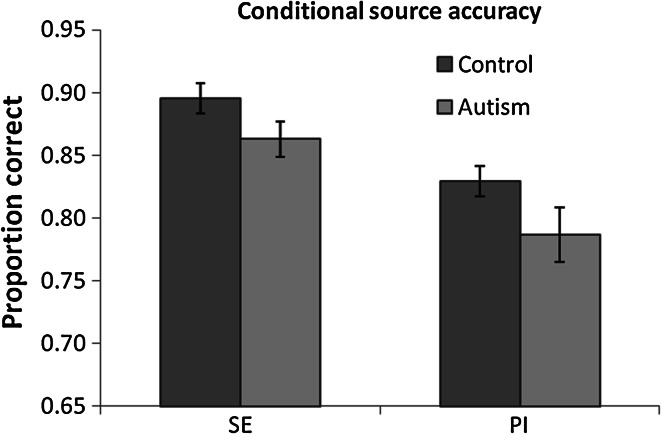
Mean proportion of recognised words for which the correct source was identified for the autism and control groups in the self-experimenter (SE) and perceived-imagined (PI) test conditions. *Error bars* SEM

To first compare whether source memory differed between groups and whether this varied according to test condition, a 2 group (autism, control) × 2 test condition (SE, PI) ANOVA was conducted. In this case, SE and PI source accuracy reflects how well participants could distinguish self and experimenter sources and perceived and imagined sources, respectively. Source accuracy was significantly higher in the SE condition than in the PI condition, F(1,46) = 29.71, *p* < .001, *η*^2^ = .39, an effect which did not differ between groups (F < .2, *p* > .7, *η*^2^ < .01). However, the autism group were found to have significantly lower source memory accuracy than the control group, F(1,46) = 4.43, *p* = .04, *η*^2^ = .09 (see Fig. 3). The same ANOVA was repeated using confidence and RT for source memory responses. Participants were more confident, F(1,46) = 13.66, *p* < .001, *η*^2^ = .22, and faster, F(1,46) = 214.42, *p* < .001, *η*^2^ = .82, for their SE source decisions than PI source decisions. Confidence did not significantly differ between the autism (mean = 732, std = 115) and control (mean = 779, std = 133) groups (F = 1.7, *p* = .20, *η*^2^ < .04), which did not vary between test conditions (F = 1.7, *p* = .19, *η*^2^ < .04). Similarly, RT did not differ between the autism (mean = 1.83 s, std = 0.29) and control (mean = 1.79 s, std = 0.25) groups (F < .5, *p* > .5, *η*^2^ < .01), which did not vary between test conditions (F = 1.2, *p* > .2, *η*^2^ < .03). Therefore, while overall source memory accuracy was reduced in the autism group, confidence and RT for source memory decisions did not differ between the groups.

#### Memory for Individual Sources

To investigate whether accuracy for indentifying individual sources (e.g. different proportions of correct source responses for self and experimenter words pairs) differed between groups, 2 ANOVAs were conducted, one in each test condition. A 2 (group: autism, control) × 2 (source: S, E) ANOVA in the SE condition revealed that participants were significantly more likely to correctly identify the source of experimenter-spoken word-pairs than self-spoken word-pairs, F(1,40) = 54.63, *p* < .001, *η*^2^ = .54, an effect which did not significantly differ between groups (F < 1.8, *p* = .19, *η*^2^ < .04). In a second 2 (group: autism, control) × 2 (source: P, I) ANOVA in the PI condition, neither the main effects of source nor the interaction between source and group were significant (Fs < 1, *p*s > .3, *η*^2^ < .02) (see Table 3 for mean source accuracy values).

**Table 3:** Source accuracy (proportion correct) for items encoded as self-spoken (S), experimenter-spoken (E), perceived (P), and imagined (I) during both the SE and PI test conditions: mean (std)

	Control		Autism	
	SE	PI	SE	PI
S	0.84 (0.09)	0.86 (0.08)	0.79 (0.13)	0.81 (0.10)
E	0.95 (0.05)	0.79 (0.08)	0.95 (0.06)	0.76 (0.14)
P	0.84 (0.10)	0.82 (0.11)	0.82 (0.09)	0.78 (0.12)
I	0.94 (0.05)	0.84 (0.08)	0.90 (0.07)	0.79 (0.14)

#### Effect of Encoding Condition on Source Memory

To investigate how source memory accuracy was affected by encoding condition, two ANOVAs were conducted, one within each test condition. The first 2 (group: autism, control) × 2 (encoding condition: P, I) ANOVA for SE source accuracy revealed that SE source memory was significantly higher for word-pairs that had been imagined at encoding as opposed to perceived, F(1,46) = 47.48, *p* < .001, *η*^2^ = .51, an effect that did not differ between groups (F < 1.3, *p* > .27, *η*^2^ < .03). For the PI test condition, a 2 (group: autism, control) × 2 (encoding condition: S, E) ANOVA revealed that later PI source memory was significantly higher for previously self-spoken items as opposed word pairs read by the researcher, F(1,46) = 16.34, *p* < .001, *η*^2^ = .26, an effect which also did not differ between the groups (F < .5, *p* > .5, *η*^2^ < .01) (see Table 3 for mean source accuracy values). Therefore, the pattern of source memory accuracy was similar between groups when looking at source memory across the various test and encoding conditions, also showing self-reference and generation effects.

### Metamemory

**Fig. 4:**
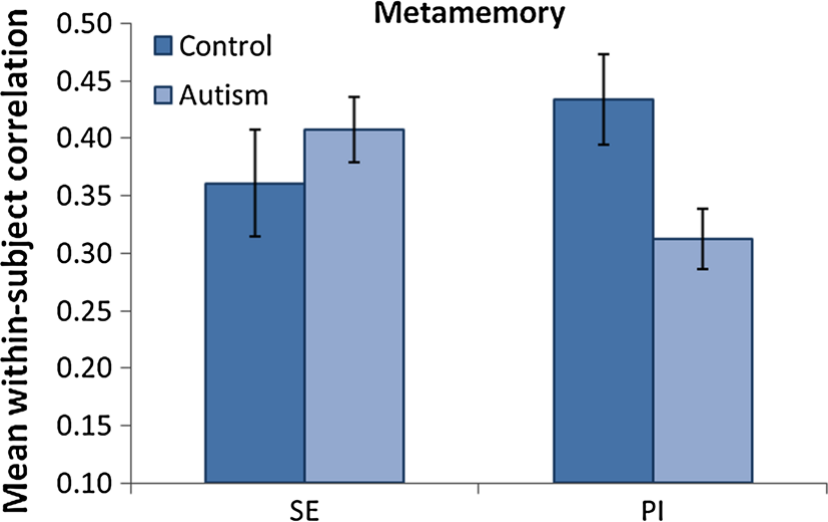
Mean within-subject correlation between confidence and accuracy as a measure of metacognitive sensitivity for source memory. *Error bars* SEM

To measure metamemory, a within-subject correlation coefficient, using Fisher’s r to z transformation, was calculated for each participant between trial-by-trial source memory accuracy and confidence. A 2 (group) × 2 (test condition: SE, PI) ANOVA on metamemory scores (see Fig. 4) revealed no difference between the SE and PI conditions (F < .1, *p* > .7, *η*^2^ < .01), and no overall difference between the 2 groups (F < 1.2, *p* > .29, *η*^2^ < .03). However, a significant interaction between group and condition, F(1,46) = 5.33, *p* = .03, *η*^2^ = .10, was due to the autism group showing significantly lower metamemory in the PI condition compared to the control group, t(46) = 2.59, *p* = .01, *d* = 0.75, but there was no difference between the groups for metamemory in the SE condition (t < 1, *p* > .39, *d* < .24). Nonetheless, metacognitive sensitivity in both conditions in both groups was greater than 0 (ts > 7.8, *p*s < .001, *d*s > 1.6).

To verify that the metamemory differences were not due to other aspects of performance that might influence the within-subject correlations (e.g. source memory accuracy, mean confidence, confidence variability), 3 low source memory performers and 3 high source memory performers from the autism and control groups respectively were removed to create 2 groups (each N = 21) that were matched on source memory accuracy (*p* > .53) and remained matched on mean confidence (*p* > .39), variability of confidence responses (*p* > .26), and all demographic variables (*ps* > .23) (see Grainger et al. 2014b for a similar approach). Repeating the ANOVA on metamemory revealed the same selective significant PI metamemory deficit in the autism group, F(1,40) = 4.33, *p* = .04, *η*^*2*^ = .09; t(40) = 2.73, *p* = .01, *d* = 0.79, and no difference in SE metamemory (t < 1, *p* > .5, *d* < 0.16) compared to the control group. Therefore, a deficit in PI metamemory in the autism group seems to be somewhat dissociable from their overall source memory deficit, as further suggested by a lack of correlation between these two scores (r = .23, *p* = .29).

## Discussion

The current study tested reality monitoring in adults with autism, with the aim of resolving previous inconsistent findings by directly contrasting two types of reality monitoring which are considered to differ with regard to underlying mentalizing processes within the same task. We also assessed the effect of self-referential processing on both recognition and source memory in autism, to test whether difficulties processing information in relation to the self may contribute to memory impairments in autism. Lastly, we assessed metamemory in autism to determine whether previously documented metamemory impairments extend to source memory and, thus, whether individuals with autism have a difficulty differentiating and monitoring internal and external details of their memories. Both groups exhibited an equal benefit of self-referential processing and imagining on later recognition relative to other encoding conditions. However, the autism group were impaired at remembering the source of studied word-pairs, an effect which did not differ according to whether self-other or perceived-imagined source discriminations were tested. Furthermore, the pattern of source responses did not differ between the groups and, as for recognition, both groups showed a benefit of self-referential processing and generation on subsequent source memory. Finally, an analysis of metamemory revealed that the autism group exhibited intact metamemory for self-other source discriminations but reduced metamemory for perceived-imagined source discriminations, indicating that the ability to monitor the accuracy of perceptual and cognitive details of source memory may be impaired in autism.

The finding that self-related encoding processes benefitted both subsequent recognition and source memory in autism is inconsistent with the view that autism is accompanied by atypical self-referential processes (Lombardo and Baron-Cohen 2011). Rather, it suggests that individuals with autism are able to use the self as an effective organisational encoding strategy, an aspect of memory that has been thought to be impaired (Crane et al. 2009). A reduced effect of the self on memory has not been demonstrated consistently in autism, with some studies reporting a reduced benefit of the self on subsequent memory in autism (Henderson et al. 2009; Lombardo et al. 2007) and others indicating an intact benefit of self-related encoding (Grainger et al. 2014a; Lind and Bowler 2009; Williams and Happé 2009). Therefore, one interpretation of the results from the current study is that self-related encoding can enhance subsequent memory in autism to the same degree as in typical controls. Alternatively, an account that may be more likely to explain the current findings involves the possibility of a distinction between the ‘psychological’ self and the ‘physical’ self in autism, with the former being impaired and the latter intact (Uddin 2011). The aforementioned studies reporting a reduced influence of the self have primarily used conceptual encoding tasks (such as “does this adjective describe you?”), whereas studies observing a benefit of the self on memory, including the current study, have used action-based encoding tasks (such as “say this word out loud”). A dissociation between ‘self-reference’ and ‘self-enactment’ has been proposed to explain these findings (Lind 2010; Williams 2010). Future research should, therefore, compare source memory for self-oriented conceptual and action-based contexts to directly test the influence of the self on episodic memory in autism.

However, even if conceptual self- processing were disproportionately impaired in autism, it would seem unlikely to be able to fully account for the reality monitoring impairment found in the current study, where source memory for word-pairs was reduced to a similar degree regardless of whether the source discrimination was self-other or perceived-imagined. The source memory deficit observed here may help to resolve previous inconsistent reality monitoring findings in autism (e.g. Lind and Bowler 2009, Grainger et al. 2014a), confirming that reality monitoring impairments do exist, even though the effect may evident only in particularly sensitive tasks. This heterogeneous reality memory impairment also has another implication, namely that mentalizing, considered particularly important for the discrimination of self-other information (Simons et al. 2008), may not fully account for the reality monitoring deficit found in autism. This interpretation is supported by evidence from Lind and Bowler (2009) who, like in the current study, observed a self-other reality monitoring deficit in autism in the presence of a self-enactment effect and, interestingly, reality monitoring ability in autism did not relate to performance on a separate mentalizing task, suggesting a dissociation between reality monitoring and mentalizing processes in autism. Further evidence comes from other studies of source memory and recollection reporting an impairment in autism that have not involved reality monitoring conditions, instead focusing on retrieval of spatial, temporal, and visual context (Bowler et al. 2004, 2014; Massand and Bowler 2013), although it is worth noting that evidence of a deficit in visual-spatial source memory in autism has not always been observed consistently (Bowler et al. 2015; Souchay et al. 2013). Nonetheless, the deficits in source memory reported here are consistent with findings from other types of tasks measuring the ability of individuals with autism to recollect context information, such as a reduction in ‘remember’ responses when recognising words or objects (Bowler et al. 2007; Cooper et al. 2015; Meyer et al. 2014) and reduced specificity of autobiographical memory (Lind and Bowler 2010; Maister et al. 2013), further illustrating memory deficits that appear to extend beyond self-referential and social processes.

An overall source memory impairment could perhaps suggest generalised PFC dysfunction in autism, although it is important to note that the link between a reality monitoring deficit and PFC dysfunction in autism can only be indirectly speculated upon based on the current study. However, this possibility seems reasonably likely considering the importance of the PFC in source memory (Mitchell and Johnson 2009), with functional specialization within this area of the brain proposed to reflect several distinct processes that contribute to source memory retrieval (Dobbins et al. 2002; Fletcher and Henson 2001). Another possibility, which has been more widely advocated in recent years, is that autism is characterised by reduced long-range connectivity between prefrontal and posterior regions (Courchesne and Pierce 2005; Just et al. 2012) which would indirectly impair frontal functions, such as monitoring and integrating information in memory. Due to the importance of the PFC for source memory, and episodic memory in general, future research should aim to study the PFC and its connectivity to other regions of the episodic memory network (see Mitchell and Johnson 2009) in autism to establish the neural correlates of impaired source memory and to investigate the specific cognitive processes, possibly supported by the PFC, that might contribute to source memory impairments. Although, it cannot be directly inferred that the same brain regions or networks will underpin the same memory functions in typical individuals and individuals with autism.

The present finding of impaired metamemory in the PI test condition in autism also fits well with a source memory deficit, further supporting the notion of a difficulty monitoring information within memory. This is the first study to demonstrate impaired metamemory for source information in autism, extending previous findings of atypical feeling-of-knowing (FOK) judgements in autism (Grainger et al. 2014b; Wojcik et al. 2013). Although both retrospective confidence judgements and prospective FOK judgements measure metamemory, evidence suggests they may be functionally and neurally dissociable (Fleming and Dolan 2012). Therefore, the observation that adults with autism also exhibit retrospective metamemory deficits for source memory judgements extends our knowledge concerning metacognitive awareness in this population. It is important to note, however, that the autism group only exhibited impaired metamemory in the PI condition and not the SE condition, a distinction that was not predicted. One reason for this difference may be the relative difficulty of the source discriminations; both groups found the SE sources easier to identify than the PI sources, meaning that evidence for SE source memory decisions was likely to be easier to monitor. Once source details become more overlapping and harder to differentiate as might be the case for PI sources, the ability of individuals with autism to monitor the accuracy of their memories might reduce. Alternatively, preserved metamemory in the self-other condition may have been attributable to intact action-monitoring, as previously discussed, whereas the perceived-imagined source condition predominantly relied on consideration of perceptual and cognitive details independent of agency. It is therefore important for future studies to test metamemory in autism within different types of context, similarity, and difficulty, for example, to clarify exactly when metamemory is impaired in autism.

The confidence-based metamemory deficits observed here are, however, in line with findings from an autobiographical memory study in autism which found that participants with autism rated their own memories as less salient and coherent (Lind et al. 2014), possibly suggesting a reduction in the subjective quality of episodic memory, with difficulty visualizing and monitoring memory details. In support of this proposal, there is evidence that metamemory deficits in autism may be characterised by underconfidence in correct memories (Grainger et al. 2014b). Conversely, there was no overall reduction in confidence for source memory in the current study, perhaps suggesting that monitoring and accessing information during retrieval might be impaired rather than the quality of the memory once retrieved. The source monitoring framework assumes that recollection is a graded process (Mitchell and Johnson 2009); therefore, research in autism would benefit from investigating both access to and the quality of episodic memories in autism rather than using traditional ‘all-or-none’ methods such as a binary choice between two sources or ‘remember’ versus ‘know’ judgements. Future studies could explicitly test the quality of recollected information in autism by adapting source memory tasks to assess ‘partial’ source memory (e.g. Dodson et al. 1998), or the ‘precision’ with which memories are recollected (Harlow and Yonelinas 2014). Future research should also focus on developing specific teaching methods and learning strategies to ameliorate the source memory and monitoring deficits provided here, possibly via the use of structured retrieval cues and minimising memory load.

In conclusion, this study investigated reality monitoring for two different types of source discrimination, self-other and perceived-imagined, in adults with autism. The autism group exhibited a reduction in reality monitoring for both types of source discrimination, which was accompanied by a deficit in metamemory when evaluating visual-perceptual and cognitive sources. These results imply that impaired monitoring and attention switching may play a role in source memory deficits in autism. Due to the link between reality monitoring, source memory, and the prefrontal cortex in the typical population, one possibility is that the source and metamemory deficits in autism could arise due to prefrontal dysfunction or reduced prefrontal-posterior connectivity. However, further research would be needed to directly test this association, which, alongside qualitative aspects of recollection, is an important area of episodic memory to investigate in autism.

## Appendix

**Table:**

Batman and Robin	home and away	cup and saucer	before and after
doom and gloom	rest and relaxation	sage and onion	ladies and gentlemen
wear and tear	mum and dad	map and compass	Slug and Lettuce
live and learn	run and hide	Winnie and Pooh	ketchup and mustard
holly and ivy	bright and early	shoes and socks	dustpan and brush
to and fro	Baskin and Robbins	Mario and Luigi	bangers and mash
Alliance and Leicester	cuts and bruises	pestle and mortar	Rosie and Jim
wine and dine	plus and minus	arts and crafts	Sonny and Cher
Jekyll and Hyde	duck and cover	fast and furious	north and south
Oxford and Cambridge	father and mother	alpha and beta	Pride and Prejudice
Mickey and Minnie	wait and see	toss and turn	dazed and confused
winners and losers	Tarzan and Jane	bacon and eggs	Posh and Becks
on and off	Fred and Ginger	day and night	give and take
crime and punishment	thrills and spills	bits and pieces	Hansel and Gretel
tweedledum and tweedledee	hot and cold	Jack and Jill	Holmes and Watson
foot and mouth	master and commander	slip and slide	ping and pong
life and death	lost and found	doctors and nurses	stocks and shares
brother and sister	heart and soul	twist and shout	law and order
loud and clear	health and safety	William and Kate	Sylvester and Tweety
Aladdin and Jasmine	Romeo and Juliet	Puss and Boots	cigarettes and alcohol
rhubarb and custard	boys and girls	birds and bees	nook and cranny
bubble and squeak	Will and Grace	Wallace and Gromit	Shrek and Donkey
town and gown	Dumb and Dumber	one and only	stars and stripes
east and west	rum and coke	push and pull	hope and glory
tried and tested	park and ride	sticks and stones	yin and yang
Anthony and Cleopatra	Gonville and Caius	safe and sound	odds and ends
rhyme and reason	rock and roll	Spongebob and Squarepants	look and listen
cowboys and indians	cash and carry	apples and pears	guys and dolls
skin and bone	this and that	Barbie and Ken	nuts and bolts
heaven and earth	tit and tat	open and shut	back and forth
fish and chips	long and short	tortoise and hare	Mitchell and Webb
terms and conditions	first and foremost	Dolce and Gabbana	fingers and toes
Pimms and lemonade	yes and no	time and again	Morecambe and Wise
Coca and Cola	black and white	drum and bass	husband and wife
rise and fall	suited and booted	strawberries and cream	Lennon and McCartney
French and Saunders	fools and horses	sugar and spice	fruit and veg
fun and games	Ross and Rachel	parsley and thyme	arm and leg
left and right	bride and groom	now and then	drink and drive
salt and pepper	Marks and Spencer	Thelma and Louise	bread and butter
Donald and Daisy	Simba and Nala	Brad and Angelina	crash and burn
meet and greet	fame and fortune	Dorothy and Toto	Punch and Judy
Mercedez and Benz	null and void	Lilo and Stitch	lock and key
cops and robbers	hints and tips	kit and kat	Chandler and Monica
Starsky and Hutch	pros and cons	Dastardly and Muttley	Humpty and Dumpty
done and dusted	sense and sensibility	Itchy and Scratchy	dead and buried
rhythm and blues	Ant and Dec	mind and body	Abercrombie and Fitch
topsy and turvy	fair and square	war and peace	trial and error
ebony and ivory	jelly and icecream	Mary and Joseph	Bosnia and Herzegovina
bat and ball	Beavis and Butthead	Guns and Roses	Procter and Gamble
Johnson and Johnson	mix and match	good and bad	dawn and dusk
Ernst and Young	meat and potatoes	king and queen	shirt and tie
Crabtree and Evelyn	Charles and Diana	spaghetti and meatballs	stand and deliver
David and Goliath	aches and pains	snakes and ladders	knife and fork
in and out	high and low	Tom and Jerry	diamonds and pearls
